# Eugenol: A promising therapeutic terpenoid against ischemia-reperfusion injury

**DOI:** 10.17179/excli2025-9036

**Published:** 2026-01-19

**Authors:** Puneet Kaur Randhawa, Deepti Gupta, Mohd Hanifa, Bivek Bajgai, Sorabh Sehajpal, Sarbeshwar Jaggi, Anjana Bali

**Affiliations:** 1 Department of Pharmaceutical Sciences, Amritsar Group of Colleges, Amritsar, Punjab, 143001, India; 2 Division of Metabolic and Cardiovascular Sciences, Burnett School of Biomedical Sciences, College of Medicine, University of Central Florida, Orlando, FL, 32827, USA; 3 Kelly Education, Orlando, USA; 4 Laboratory of Neuroendocrinology, Department of Pharmacology, Central University of Punjab, Ghudda, Bathinda, India; 5 Simon Fraser University, Burnaby, BC, Canada

**Keywords:** Eugenol, Ischemia-reperfusion injury, AMPK, PI3K, Nrf2, ACE

## Abstract

Ischemic disorders are one of the prime causes of mortality and disability among various individuals across the globe. Although drug treatment/percutaneous coronary interventions may recanalize the obstructed blood vessels, yet reperfusion therapy may aggravate tissue damage and result in ischemia-reperfusion injury. Eugenol, a phenolic monoterpenoid (4-allyl-2-methoxyphenol), has been used extensively in various preclinical studies as an antioxidant compound that ameliorates ischemia-reperfusion injury in several organs, including the heart, brain, kidney, and intestine. This protective effect of eugenol is attributed to its ability to influence various several key signaling pathways. These include the AMPK-mTOR-P70S6K (AMP-activated protein kinase-mammalian target of rapamycin-p70 ribosomal S6 kinase) pathway, AMPK/GSK3β (Glycogen synthase kinase-3 beta) axis, PI3K/Akt (phoshatidylinositol-3 kinase/ protein kinase B) signaling, which help to mitigate oxidative damage and inflammation. It also modulates the activity of the Nrf2 transcription factor, ACE, and the apoptotic pathway, affects histone acetylation, and alters the expression of HMGN1, PPP2Ca, and CD151 genes, demonstrating its wide-ranging therapeutic effects. In this review, we will discuss the preclinical evidences and potential mechanisms of action of eugenol-dependent protective benefits against ischemia-reperfusion injury.

See also the graphical abstract(Fig. 1).

## 1 Introduction

The prevalence of ischemic diseases globally has been increasing alarmingly in the past few years (Sun et al., 2020[44]; Randhawa et al., 2023[37]). Ischemia-reperfusion injury is a dynamic phenomenon caused by prolonged ischemia followed by reperfusion, which may result in reversible or irreversible damage to the tissue (Randhawa and Jaggi, 2018[36]). It predominantly affects oxygen-dependent organs, including the liver, brain, heart, kidneys, stomach, and intestine (Akbari et al., 2018[4]). Generally, the incidences of ischemia-reperfusion injury are higher in clinical settings in patients suffering from myocardial infarction, stroke, or the ones undergoing surgical interventions, for instance, organ transplantation, coronary angioplasty, and reperfusion after thrombolytic therapy (Randhawa et al., 2015[35]). Therefore, there is a dire need to explore an emphatic molecule that may ameliorate ischemia-reperfusion-induced injury.

Eugenol is a bioactive, antioxidant compound widely known for alleviating ischemia-reperfusion injury in various organs, including the heart, liver, brain, kidney, and intestine (Mnafgui et al., 2016[27]; Ahmad et al., 2018[3]; Wang et al., 2021[49]). Various researchers have established eugenol's role in modulating the myocardium's activity (Feng et al., 2015[17]; Mnafgui et al., 2016[27]; Sedighi et al., 2018[42]). Sedighi and co-workers revealed that eugenol could combat arrhythmia and exert a cytoprotective effect on the heart (Sedighi et al., 2018[42]). Eugenol pretreatment limited the ventricular remodeling process in the myocardium and improved the primary outcome parameters in a rat model of ischemia-reperfusion injury (Mnafgui et al., 2016[27]). Intriguingly, a study unveiled that eugenol administration also reduced cerebral ischemia-reperfusion-induced injury and improved neurological scores in rats (Sun et al., 2020[44]). Furthermore, supplementation of eugenol modulated the extent of ischemia-reperfusion injury in other organs, including the liver (Abd El Motteleb et al., 2014[1]), intestine (Saleh and El-Shorbagy, 2017[40]), and kidney (Kuang et al., 2023[22]). Apart from this, eugenol exhibits anti-cancer, anti-diabetic, anti-bacterial, anti-fungal, anti-viral, anti-nociceptive, anti-pyretic, and anti-inflammatory effects (Nisar et al., 2021[28]; Taleuzzaman et al., 2021[45]; Begum et al., 2022[7]).

**Figure 2:**
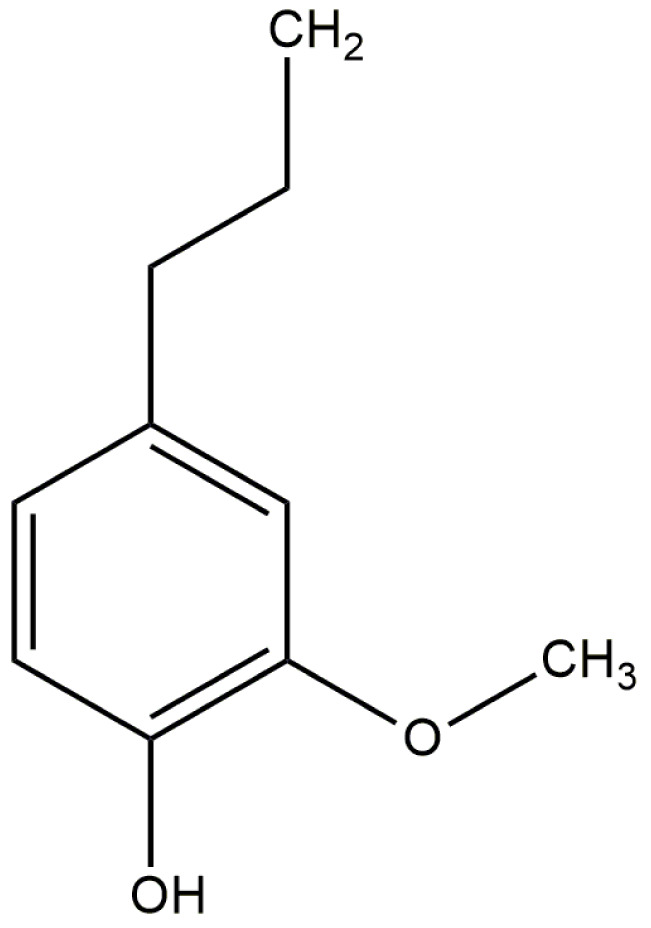
Chemical structure of eugenol

Eugenol is a phenolic monoterpenoid (4-allyl-2-ethoxyphenol) (Figure 2(Fig. 2)) which is chiefly found in a wide range of plants including Syzygium aromaticum (clove), Zingiber officinale (ginger), Cinnamomum verum (cinnamon), Ocimum basilicum (basil), Myristica fragrans (Nutmeg), etc (Nisar et al., 2021[28]; El-Far et al., 2022[15]). Eugenol is usually a colourless or yellowish coloured liquid which is slightly soluble in water but easily soluble in organic solvents. It is exclusively isolated from clove bud, stem, and leaves (Nisar et al., 2021[28]; Ulanowska and Olas, 2021[48]). Eugenol can also be obtained synthetically by guaiacol allylation with allyl chloride or via a biotransformation process using Escherichia coli, Corynebacterium sp., and Bacillus cereus (Nisar et al., 2021[28]). Apparently, Syzygium aromaticum is native to Indonesia but is cultivated in several regions across the globe (Cortés-Rojas et al., 2014[13]). Clove is one of the richest sources of phenolic compounds such as eugenol, eugenol acetate, and gallic acid, such that it suitable for the pharmaceutical, cosmetic, and agricultural industry (Cortés-Rojas et al., 2014[13]; Ulanowska and Olas, 2021[48]). In fact, the WHO has declared Eugenol as generally recognized as safe (GRAS) and a nonmutagenic substance (Nisar et al., 2021[28]). Eugenol has a bioavailability score (measure of drug availability at the site of action) of 0.55, which is considered moderately satisfactory for exogenous drugs (Begum et al., 2022[7]). The n-octanol and water ratio, or the LogP value for eugenol, is 2.1293, which indicates it is a lipophilic compound. Also, the polar surface area, which is a measure of cell membrane permeability, was 72.109 Ǻ squared, suggesting that it can easily cross the plasma membrane barrier. Notably, eugenol exhibited good permeation through the skin as well as the intestinal membrane, indicating that it can be administered via oral or topical routes (Lipinski et al., 2001[24]; Begum et al., 2022[7]). Studies on healthy human volunteers have shown that it is rapidly metabolized owing to its short half-life and has minimal risk of accumulation in any body tissue. Eugenol is regarded as a nontoxic molecule, and its maximum tolerated level for humans was 1.024 (mg/kg/day) (Begum et al., 2022[7]). Eugenol can be encapsulated with several carriers, such as solid lipid nanoparticles (Garg and Singh, 2011[18]), liposomes (Sebaaly et al., 2015[41]), and glycodendritic polyamine dextran (Singh et al., 2016[43]) to achieve the targeted drug delivery in normal or diseased animal models. However, its usage in comorbid conditions requires further studies to validate its efficacy and potency.

Mechanistically, eugenol is known to shield various tissues against ischemia-reperfusion injury via modulating various signaling pathways, including the AMPK-mTOR-P70S6K pathway, AMPK/GSK3β axis, PI3K/Akt signaling, limiting oxidative injury, manipulating inflammatory, Nrf2 (nuclear factor erythroid 2-related factor 2), ACE (angiotensin converting enzyme), apoptotic pathway, acetylation of histone proteins, and expression of HMGN1 (High Mobility Group Nucleosome Binding Domain 1), PPP2Ca (Protein Phosphatase 2 Catalytic Subunit Alpha), and CD151 genes. In this review, we shall describe the role of eugenol in mitigating ischemia-reperfusion injury and the signaling process involved in limiting the damage.

## 2 Preclinical Evidences of Eugenol-Mediated Effects against Ischemia-Reperfusion Injury in Different Organs

### 2.1 Heart

Ischemia and, after that, reperfusion during conditions like acute myocardial infarction or surgical intervention may damage the myocardium (Singh et al., 2016[43]). Preclinically, myocardial infarction is induced either via ligation of the coronary artery (Ovsepyan et al., 2011[30]; De Villiers and Riley, 2020[14]) or via administering a high dose of isoproterenol (Pipaliya and Vaghasiya, 2012[33]; Sajid et al., 2022[39]). Isoproterenol is a synthetic catecholamine (β-adrenergic agonist) that can be administered non-invasively and efficiently to induce myocardial infarction in experimental animals (Patel et al., 2010[31]; Liu et al., 2022[25]; Sajid et al., 2022[39]). Studies have shown that isoproterenol induces myocardial injury similar to that produced by ischemia in the rodent heart, indicating that isoproterenol induces myocardial hypoxia or ischemia to induce cardiac injury (Brooks and Conrad, 2009[8]; Allawadhi et al., 2018[5]; Pham et al., 2023[32]). Evidently, isoproterenol treatment significantly decreased peak systolic blood pressure in mice, suggesting that the injury might be mediated by hypoperfusion (Pham et al., 2023[32]). In fact, a study found that the isoproterenol-induced myocardial injury model is more pertinent to myocardial ischemia caused due to excessive compensatory overactivity of the sympathetic nervous system (Liu et al., 2022[25]). Isoproterenol plausibly causes severe damage to the cardiac myocytes because of calcium overload, myocardial hyperactivity-induced hypoxia, coronary hypotension, exhaustion of energy reserve, and exacerbated release of free radicals (Patel et al., 2010[31]; Sajid et al., 2022[39]). The pathophysiological changes produced after isoproterenol administration are analogous to those happening in human myocardial alterations (Rajadurai and Stanely Mainzen Prince, 2007[34]; Sajid et al., 2022[39]).

Various scientists have brought insight into the role of eugenol in alleviating ischemia-reperfusion injury in the myocardium (Mnafgui et al., 2016[27]; Feng et al., 2018[16]; Sedighi et al., 2018[42]). Mnafgui and co-workers have reported that isoproterenol administration induced myocardial infarction in the rats in terms of alteration in ECG pattern, upsurge in heart weight index, increase in the troponin T, CK-MB (creatine kinase-myoglobin binding), and LDH (Lactate Dehydrogenase) level in the serum. Also, isoproterenol treatment produced myocardial necrosis, infiltration of inflammatory cells, and deteriorated hemodynamic function in the rats. In addition, inflammatory biomarkers such as fibrinogen, α1, α2, β1, β2, and γ globulins were significantly increased, whereas albumin was considerably reduced in the infarcted hearts. Besides, isoproterenol treatment escalated ACE activity in the plasma, kidney, and heart. However, eugenol administration (50 mg/kg for seven days) significantly reduced the markers of cardiac injury in the serum, decreased inflammatory proteins, and remarkably improved the activity of superoxide dismutase and glutathione peroxidase in the heart. Eugenol administration limited the generation of thiobarbituric acid-reactive substances. Altogether, eugenol administration modulated oxidative stress, reduced the markers of cardiac injury, and prevented cardiac remodeling in the infarcted heart (Mnafgui et al., 2016[27]).

Sedighi et al. have reported that oral or intragastric administration of ethanolic extract of *Cinnamomum zeylanicum* (50, 100, or 200 mg/kg) for 14 consecutive days protected the heart against sustained ischemia-reperfusion injury in an *in vivo* rat model of regional heart ischemia. The authors found that the administration of ethanolic cinnamon bark extracts considerably improved ischemia-reperfusion-induced myocardial injury in terms of reduction of infarct size compared to the control. Also, the authors found that the administration of ethanolic extract mitigated ventricular tachycardia and episodes of ventricular ectopic beats during ischemia in comparison to the control. Moreover, the extract stabilized the ST segment changes, QTc shortening, and improved R-wave amplitude and heart rate during prolonged ischemia. Apart from this, administration of the extract remarkably elevated superoxide dismutase and glutathione peroxidase activity.

Interestingly, serum cardiac troponin I, LDH, and malondialdehyde (MDA) levels significantly decreased in the serum five days after reperfusion. The HPLC analysis revealed the presence of cinamic acid, methyl eugenol, and cinnamaldehyde in the ethanolic extract, and their amounts were 8.99 ± 0.5, 13.02 ± 1.8, and 14.63 ± 1.1 mg/g, respectively. Apparently, methyl eugenol accounts for ~35 percent of the total protective effect. Altogether, the ethanolic extract of cinnamon bark shielded the heart against ischemia-reperfusion injury, possibly due to its antioxidant effect (Sedighi et al., 2018[42]).

Intriguingly, Feng et al. have reported that intraperitoneal delivery of eugenol (20 mg/kg/day) in rats significantly limited cardiac troponin I, CK-MB, TNF-α, interleukin-6 (IL-6) levels in the serum, and MDA content in the transplanted heart. The authors identified that eugenol treatment limited the irregular arrangement of cardiac muscle fibers, infiltration of inflammatory cells, and development of interstitial edema. Also, eugenol treatment reduced TUNEL (Terminal deoxynucleotidyl transferase dUTP nick end labeling)-positive muscle cells. The study indicated that eugenol reduced myocardial apoptosis and alleviated myocardial injury to shield the transplanted heart (Feng et al., 2018[16]) (Table 1(Tab. 1); References in Table 1: Abd El Motteleb et al., 2014[1]; Feng et al., 2018[16]; Kuang et al., 2021[21]; Mnafgui et al., 2016[27]; Saleh and El-Shorbagy, 2017[40]; Sedighi et al., 2018[42]; Sun et al., 2020[44]; Wang et al., 2021[49]; Won et al., 1998[50]). Interestingly, eugenol also has the potential to inhibit tetrodotoxin-sensitive and resistant Na^+^ (sodium) currents in a concentration-dependent manner (Kd value 308 µM, 543 µM) but independent of the frequency of depolarization stimulations. Eugenol administration didn't influence activation of Na^+^ currents, and eugenol interacted mainly with resting and inactivated Na^+^ channels (Cho et al., 2008[10]). Another study found that eugenol inhibits voltage-gated Na^+^ channels to exhibit anti-arrhythmic effects (Teixeira-Fonseca et al., 2021[46]). This is further corroborated by the fact that eugenol administration to an *ex vivo* heart preparation and isolated ventricular cardiomyocytes (guinea pig) shifted the stationary inactivation curve towards left and delayed the recovery of Na^+^ channels from inactivation state, indicating its anti-arrhythmic potential (Teixeira-Fonseca et al., 2023[47]). There is a dire need to study ligand-channel interaction using advanced models and tailor the dosage for the individuals to limit life-threatening complications.

### 2.2 Liver

Liver injury, tumor resection, or liver transplantation may result in liver ischemia/reperfusion (I/R) injury, which may damage the liver (Liu et al., 2019[26]). Abd El Motteleb and co-workers reported that occlusion of the blood vessels and bile ducts supplying the left and median liver lobes using a vascular clamp for 45 minutes and then releasing the clamp to induce reperfusion led to ischemia-reperfusion injury in the rats (Abd El Motteleb et al., 2014[1]). Interestingly, the authors found that oral administration of eugenol (10 mg/kg/day) for 15 days before exposure to sustained ischemia safeguarded the liver against ischemia-reperfusion injury. The protective effect was observed in terms of considerable reduction in serum ALT (alanine transaminase), AST (aspartate aminotransferase), LDH levels, hepatic MPO (Myeloperoxidase), and improvement in the hepatic architecture as well as function. Besides, the authors have reported that eugenol treatment reduced hepatic MDA content and increased GSH (Glutathione) levels to boost the antioxidant status of the host (Abd El Motteleb et al., 2014[1]). However, the authors found that treatment with a higher dose of eugenol (100 mg/kg/day) considerably amplified oxidative, inflammatory, and apoptotic markers compared to ischemia-reperfusion-induced injury in rats. Altogether, the authors indicated that eugenol could elicit bifurcated responses in the host based on the dose employed. The lower dose exhibits a protective response in the liver via modulating the oxidative status and limiting ischemia-reperfusion injury in the rats (Abd El Motteleb et al., 2014[1]).

Recently, Wang and co-workers have reported that administration of methyl eugenol ameliorated ischemia-reperfusion injury in C57BL/6J mice subjected to ischemia (60 minutes), followed by reperfusion (6 hours). The authors found that methyl eugenol also attenuated hypoxia-reoxygenation injury in AML12 (a mouse liver cell line) cells subjected to hypoxia (24 hours), followed by normoxia (18 hours). On the whole, methyl eugenol abrogated ischemia-reperfusion-induced injury, inflammatory response, and apoptosis in both *in vitro* and *in vivo *models (Wang et al., 2021[49]) (Table 1(Tab. 1)).

### 2.3 Brain

Cerebral ischemia-reperfusion injury accounts for long-term disability among various individuals and severely threatens the health and the economy worldwide. There is a dire need to search for an effective treatment strategy to ensure the recovery of ischemia-reperfusion in patients and improve their quality of life (Sun et al., 2020[44]). A study by Won et al. reported that eugenol treatment (50, 100, 200 mg/kg, i.p.) in gerbils immediately after occlusion (5 min) of the bilateral carotid arteries under free-regulated temperature preserved neuronal cells in the CA1 (Cornu Ammonis 1) region after ischemic insult (7 days). However, this protective effect was not seen in the gerbils' thermoregulated condition, indicating that eugenol elicits a neuroprotective effect against ischemic damage via exhibiting hypothermic action (Won et al., 1998[50]). A group of researchers has developed eugenol nanoparticles for the intranasal administration of eugenol to treat cerebral ischemia (Ahmad et al., 2018[3]).

Choi et al. documented that administration of methyl eugenol considerably reduced cerebral ischemic injury in the middle cerebral artery occlusion model (MCAO; occlusion of the artery for 1.5 hours and reperfusion for 24 hours). Also, the treatment of cultured cerebral cortical neurons with methyl eugenol in oxygen-glucose-deprived conditions (1 hour) and subsequent re-oxygenation (24 hours) remarkably reduced cell death (Choi et al., 2010[11]). Sun and co-workers have reported that eugenol treatment salvaged the rat brain against cerebral ischemia-reperfusion injury in MCAO. The authors found that administering eugenol and rapamycin (an autophagy activator) considerably improved neurological deficit, infarct volume, brain water content, and apoptosis in the MCAO rat model. However, this neuroprotective effect was abolished in the presence of 3-MA (an autophagy inhibitor), indicating that the protection was exhibited by enhancing autophagy. Likewise, using HT22 cells, eugenol treatment increased cell viability and prohibited apoptosis in oxygen-glucose-deprived/reperfused cells (Sun et al., 2020[44]) (Table 1(Tab. 1)).

### 2.4 Intestine

Intestinal disease, distant organ failure, or surgical procedure may restrict intestinal blood flow to induce intestinal injury, which is further exaggerated after reperfusion (Gonzalez et al., 2015[19]). Saleh and El-Shorbagy have reported that administration of methyl eugenol prior to intestinal ischemia (30 minutes) and subsequent reperfusion (60 minutes) protected against sustained intestinal ischemia-reperfusion injury. Pretreatment with methyl eugenol considerably reduced serum LDH and tissue MDA levels and improved the small intestine's architecture (Saleh and El-Shorbagy, 2017[40]) (Table 1(Tab. 1)).

### 2.5 Kidney

Ischemia-reperfusion injury in the kidney usually occurs when the organ's blood flow is compromised during transplantation or while undergoing a surgical procedure (Randhawa et al., 2015[35]). The susceptibility and outcome of exposure to the ischemic condition may depend on the exposed glomerular cell type (epithelial/endothelial) (Chatauret et al., 2014[9]; Randhawa et al., 2015[35]). Very recently, a group of researchers reported that methyl eugenol (20 mg/kg, i.p) treatment in mice for five consecutive days ahead of ischemia-reperfusion surgery considerably ameliorated renal destruction and promoted the survival rate of renal cells. Also, the authors found that methyl eugenol limited oxidative stress and inhibited mitochondrial lesions in the mice subjected to acute kidney injury. In vivo methyl eugenol treatment ameliorated the development of renal fibrosis during the nonfatal ischemia-reperfusion cycle. Apart from this, *in-vitro *exposure of methyl eugenol (40 μmol/L) treated proximal tubule epithelial cells to hypoxia-reoxygenation, which led to improved survival rate and limited oxidative stress (Jiang et al., 2014[19]) (Table 1(Tab. 1)).

Ribeiro-Silva et al found that chronic administration of eugenol (2.6 mg, 5.2 mg, 7.8 mg) in the diet in combination with pulegone (2.6 mg, 5.2 mg, 7.8 mg) as well as acute treatment of eugenol (1000 mg) in combination with pulegone (1000 mg) exerted negative effects in mice. Eugenol and pulegone induced behavioral changes, specifically alteration of posture, reduced food and water consumption, causing weight loss. The authors found that eugenol and pulegone treatment increased catalase, glutathione reductase activity. Histopathological analysis revealed the presence of inflammatory infiltrates in the lungs of the treated mice. The authors indicated that eugenol administration exhibits beneficial or harmful effects based on the dosage administered and the duration of treatment (Ribeiro-Silva et al., 2022[38]).

## 3 Mechanisms Underlying Eugenol-Mediated Protective Effects against Ischemia-Reperfusion Injury

### 3.1 Inhibition of ACE activity

ACE is a crucial enzyme that metabolizes various peptides and plays a vital role in the vascular remodeling of the heart (Garg and Singh, 2011[18]). ACE profoundly regulates the cardiac renin-angiotensin system during myocardial ischemia. Previous studies have indicated that perturbations in myocardial metabolism alter ACE expression in the heart (Cohen-Segev et al., 2014[12]).

Mnafgui and co-workers found that ACE activity remarkably increased in the plasma, heart, and kidney of the isoproterenol-treated rats. Interestingly, the authors found that eugenol administration improved the cardiac biomarkers, increased the activity of superoxide dismutase and glutathione peroxidase in the heart, reduced inflammatory mediator proteins, thiobarbituric acid-reactive substances, and prevented the progression of the ventricular remodeling process via inhibition of ACE activity (Mnafgui et al., 2016[27]).

### 3.2 Inhibition of inflammation, apoptosis, and NO production

Studies have shown that heart transplantation treatment or surgical procedure induces acute inflammation and early apoptosis in the cardiomyocytes (Ostriker et al., 2021[29]; Liu et al., 2022[25]). Abd El Motteleb et al. unveiled that eugenol (10 mg/kg/day) treatment reduced TNF-α, hepatic nuclear factor-κB (NF-κB) p65, and caspase-3 expression in the serum. The authors found that eugenol protects the liver against ischemia-reperfusion injury, possibly via attenuating lipid peroxidation, down-regulating inflammatory mediators, and limiting apoptosis (Abd El Motteleb et al., 2014[1]).

**Figure 3:**
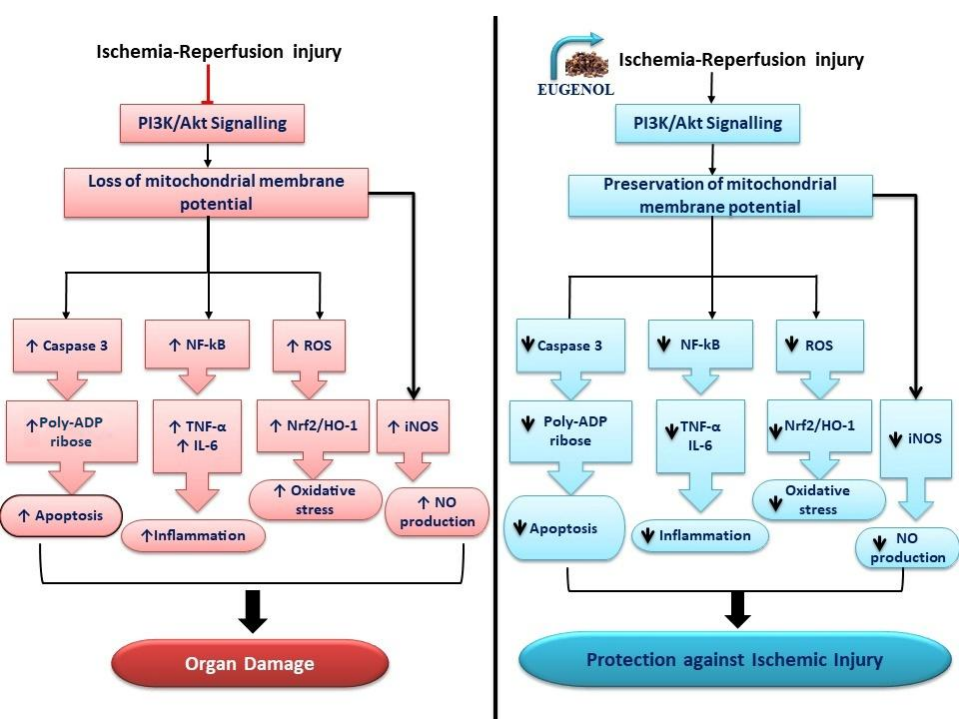
Attenuation of inflammation, apoptosis, and signaling pathway in eugenol-mediated protective effects against ischemia-reperfusion injury: Eugenol administration during ischemic conditions activates PI3/Akt signaling and results in preservation of mitochondrial membrane potential (MMP), which reduces the level of oxidative stress in the cells. The reduction in oxidative stress curbed the activation of inducible nitric oxide synthase (iNOS) and limited the production of NO and TNF-α. The reduction in TNF-α further failed to activate NF-κB, which led to reduced production of IL-6 and TNF-α. TNF-α reduction also reduced BAX activation and resulted in a reduction of apoptosis. The preservation of MMP reduced the level of caspase-3, Poly (ADP-ribose) and led to an overall reduction in apoptosis.

In corroboration with the above study, Saleh and co-workers reported that pretreatment with methyl eugenol down-regulated messenger RNA and protein expression of TNF-α and IL-6 (inflammatory cytokines) in a rat model of ischemia-reperfusion injury. Also, there was a marked reduction in the formation of apoptotic DNA fragmentation after treatment with methyl eugenol. Methyl eugenol ameliorated ischemia-reperfusion injury by limiting oxidative stress and inflammatory cytokines gene expression (Saleh and El-Shorbagy, 2017[40]). Western blot analysis unfolded the fact that eugenol administration reduced myocardial apoptosis in heterotopic transplanted hearts in terms of reduction in the expression of cleaved Poly (ADP-ribose) polymerase 1 (a hallmark of apoptotic cell death), BAX (regulator of apoptosis), active caspase-3 level (death protease) and augmentation in the expression of B-cell lymphoma 2 (anti-apoptotic) (Feng et al., 2018[16]) (Figure 3(Fig. 3)).

Choi and co-workers also found that methyl eugenol treatment reduced caspase-3 activation in cultured cerebral cortical neurons. Also, the production of superoxide ions and intracellular oxidative stress was considerably reduced in the ischemic brain. Furthermore, the administration of methyl eugenol suppressed inducible nitric oxide synthase expression and nitric oxide production. Also, methyl eugenol limited the generation of pro-inflammatory cytokines in the ischemic brain and immunostimulant mixed glial cells (Choi et al., 2010[11]). Further, Kuang and co-workers reported that methyl eugenol treatment preserved the mitochondrial membrane potential of HK-2 cells exposed to hypoxia-reoxygenation and curbed the release of reactive oxygen species (Kuang et al., 2021[21]). Thus, eugenol treatment protects the tissues against ischemia-reperfusion injury-induced damage via counteracting oxidative stress, limiting apoptosis, and production of inducible NO.

### 3.3 Modulation of intracellular signal transduction pathways

Various intracellular signal transduction pathways are dysregulated during ischemia-reperfusion injury (Aggarwal et al., 2016[2]), but the activity of these pathways can be modulated in the presence of eugenol to limit ischemia-reperfusion-induced damage to the organs (Sun et al., 2020[44]; Wang et al., 2021[49]). Indeed, a comprehensive understanding of the molecular pathways of eugenol-dependent cytoprotective effects is the key to salvaging the tissue against ischemia-reperfusion injury. MTOR/AMPK/PI3K/Akt signaling pathway plays a vital role in maintaining cellular metabolism and regulating various functions, including cell proliferation, adhesion, invasion, migration, protein synthesis, etc. (Zhang et al., 2014[51]). A dynamic relationship exists between the mTOR/AMPK pathway and autophagy such that this pathway can modulate autophagy to a large extent (Zhang et al., 2014[51]) (Figure 3(Fig. 3)).

Sun and co-workers found that eugenol treatment protected against cerebral ischemia-reperfusion injury via inducing autophagy and modulation of the AMPK/mTOR/P70S6K signaling pathway. Using western blot, the authors found that ischemia-reperfusion increased p-AMPKα/AMPKα ratio while reducing the p-mTOR/mTOR and p-P70S6K /P70S6K ratio in the brain tissue in comparison to sham. Perhaps, eugenol administration further raised p-AMPKα/AMPKα ratio while reducing p-mTOR/mTOR and p-P70S6K /P70S6K ratio in the brain compared to the ischemia-reperfusion injury group. The study indicated that eugenol treatment ameliorated cerebral ischemia-reperfusion injury by enhancing autophagy and modulating AMPK/mTOR/P70S6K signaling pathway (Sun et al., 2020[44]).

The PI3K/Akt pathway is imperative in providing cytoprotective effects against ischemia-reperfusion injury (Li et al., 2017[23]). Wang and co-workers reported that methyl eugenol ameliorated ischemia-reperfusion injury in both *in vitro* and *in vivo* models of ischemia-reperfusion injury. Methyl eugenol treatment also abrogated the inflammatory response and apoptosis in the liver ischemia-reperfusion injury model. The authors found that administration of LY294002 (PI3K/Akt signaling inhibitor) in combination with methyl eugenol ameliorated the protective effects of methyl eugenol. This indicated that methyl eugenol possibly shields against ischemia-reperfusion injury by activating the PI3K/Akt signaling pathway (Wang et al., 2021[49]).

### 3.4 Transcriptional regulation of Nrf2

Nrf2 is a defensive pathway that counters oxidative cell stress by activating many antioxidant enzymes, including heme oxygenase-1 (HO-1), glutathione peroxidase, etc. (Alonso-Piñeiro et al., 2021[6]). Kuang and co-workers revealed that methyl eugenol promoted the expression of the Nrf2/HO-1 gene and translocation of Nrf2 to the nucleus and down-regulated the expression of Nox4 to limit apoptosis in HK-2 cells. The methyl eugenol-induced protection was further reversed by ML385 (specific Nrf2 inhibitor). The study indicated that methyl eugenol effectively protects HK-2 cells against hypoxia-reoxygenation-induced injury, possibly via activation of the Nrf2/HO-1 signaling pathway and down-regulation of Nox4 (Kuang et al., 2021[21]).

The same group of researchers reported that in both *in vitro* and *in vivo *renal oxidative damage models, methyl eugenol treatment produced nuclear retention of Nrf2 in the tubular cells to reduce oxidative stress. Using molecular docking and specific inhibitors (CC and DIF-3), the authors unveiled that methyl eugenol is bound with high affinity to the AMPK binding pocket. They stimulated the AMPK/GSK3β axis, which in turn prohibited the Nrf2 nuclear export signal, resulting in nuclear retention of Nrf2 in the renal cells. Overall, the study indicated that methyl eugenol regulates the AMPK/GSK3β axis to facilitate the translocation of Nrf2 from cytoplasm to the nucleus, resulting in Nrf2 nuclear retention, which subsequently enhances antioxidant gene transcription to protect the kidneys from oxidative damage (Kuang et al., 2023[22]).

### 3.5 Modulation of H3K27ac, H3K9ac and expression of HMGN1, PPP2Ca, and CD151genes

**Figure 4:**
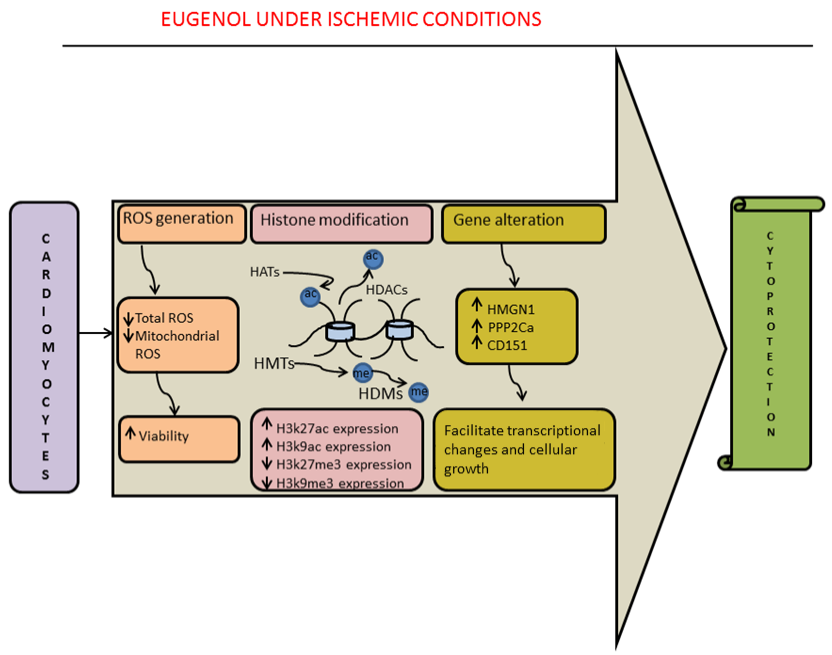
Modulation of gene expression in eugenol-mediated protective effects against ischemia-reperfusion injury: The treatment of cells with eugenol considerably reduced the level of oxidative stress and improved the viability of the cells. Eugenol treatment increased H3K27ac and H3K9ac but reduced H3K27me3 and H3K9me3, suggesting transcriptional activation in the nucleus. Also, the genes controlling cellular growth, including HMGN1, PPP2Ca, and CD151, were considerably up-regulated in the presence of eugenol under ischemic conditions. Overall, these transcriptional changes induced cytoprotective effects in the heart.

Histone acetylation or methylation are epigenetic modifications that alter the chromatin architecture and regulate the expression of genes by opening or closing the chromatin structure based on the position of the histone protein being acetylated or methylated (Randhawa et al., 2023[37]). Very recently, our study revealed that eugenol administration under ischemic conditions restored a marked reduction in the level of acetylation of histone proteins at H3K27 (histone H3 at lysine 27) and H3K9 positions (histone H3 at lysine 9; markers of transcriptional activation) but reduced methylation at H3K27 and H3K9 positions (markers of transcriptional repression) during ischemia. Importantly, the application of the HAT blocker negated the eugenol-mediated beneficial effects. Eugenol administration under ischemic conditions improved cellular viability and limited oxidative stress in the cardiomyocytes. In fact, RNA-sequence analysis revealed that during ischemia, the expression of three protein-coding genes, i.e., HMGN1, PPP2Ca, and CD151, was modulated. However, eugenol administration under ischemic conditions restored the drop in the expression of the HMGN1, PPP2Ca, and CD151 genes. Thus, our study authors indicated that restoration of HMGN1 and PPP2Ca gene expression might aid in maintaining open chromatin structure and expedite transcriptional change via acetylation of histone 3 (Randhawa et al., 2023[37]). Therefore, it could be plausible to suggest that eugenol-mediated transcriptional changes are followed by preservation of cellular homeostasis and reduced cellular hypertrophy, consequently increased cell viability (Figure 4(Fig. 4)).

## 4 Conclusion and Future Perspective

Our study unveiled that eugenol protects against ischemia-reperfusion injury in various tissues, including the heart, brain, kidney, intestine, liver, etc. Eugenol can activate various cell signaling pathways to shield several organs against ischemia-reperfusion injury. Owing to the ability of eugenol to counteract oxidative stress, it can be explored as a novel therapeutic agent to treat ischemic conditions in several organs. The potential of eugenol to prevent ischemia-reperfusion injury in many organs has been demonstrated, but further preclinical research is still needed to determine the therapeutic effects of eugenol soon after reperfusion and the optimal window of time for administration. In addition, further research in this field shall pave way for the use of eugenol as a protective agent against ischemia-reperfusion injury in a clinical setting.

## Declaration

### Conflict of interest

The authors declare no conflict of interest.

### Authors' contributions

PKR, DG and SJ collected the literature and wrote the manuscript.

MH, BB, and SS reviewed the findings and hypothesized the plausible mechanism.

AB drafted the idea, analyzed the manuscript findings, and wrote the paper.

All authors have contributed equally.

### Availability of data and materials

All the figures will be supplied in original form.

### Artificial Intelligence (AI) - assisted technology

No artificial intelligence tools were used in the preparation, writing, editing, or analysis of this manuscript.

## Figures and Tables

**Table 1:**
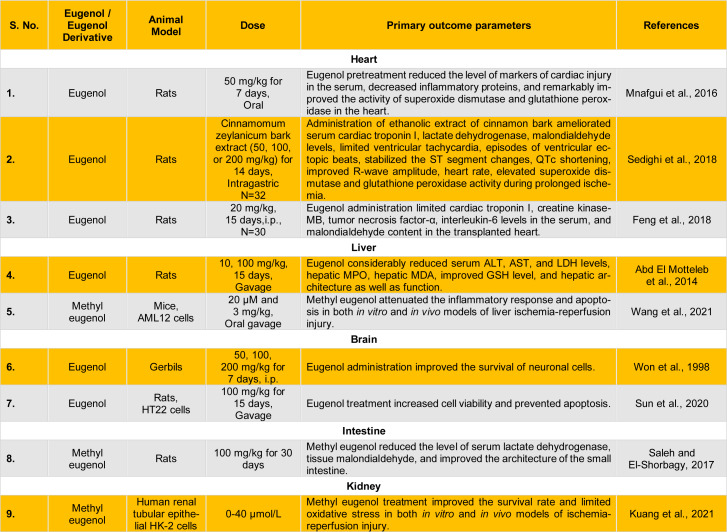
Summarized evidence of eugenol-mediated beneficial effects against ischemia-reperfusion injury in different organs

**Figure 1:**
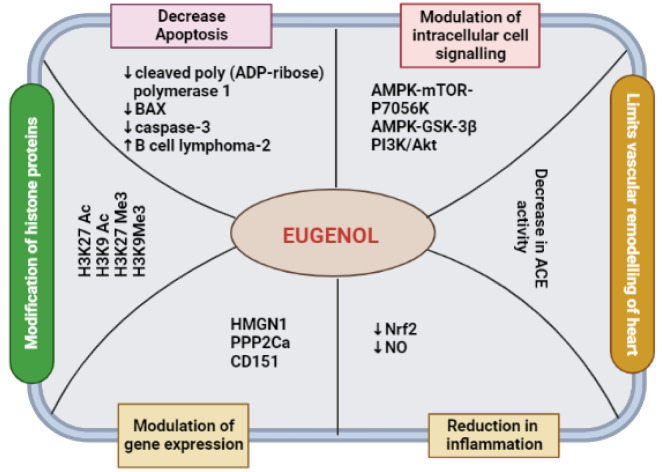
Graphical abstract
